# Early Posttraumatic Antifibrinolysis Reduces Perioperative Hidden Blood Loss in Elderly Patients with an Intertrochanteric Fracture: A Randomized Controlled Trial

**DOI:** 10.3390/jcm12155018

**Published:** 2023-07-30

**Authors:** Gang Luo, Zhiguo Chen, Jiacheng Liu, Weidong Ni, Wei Huang

**Affiliations:** Orthopedic Laboratory of Chongqing Medical University, Department of Orthopedics, The First Affiliated Hospital of Chongqing Medical University, Chongqing 400016, China

**Keywords:** posttraumatic antifibrinolysis, tranexamic acid, intertrochanteric fracture, hidden blood loss

## Abstract

Background: This study aimed to determine the efficacy and safety of posttraumatic antifibrinolysis with multidose tranexamic acid (TXA) in reducing perioperative hidden blood loss (HBL) in elderly intertrochanteric fracture patients. Method: Ninety-six elderly intertrochanteric fracture patients admitted to our department from June 2021 to September 2022 were randomized into two groups. The control group (Group A) received 100 mL of normal saline, while the experimental group (Group B) received 1.5 g of TXA intravenously q12 h from postadmission Day 1 (PAD1) to the day before surgery, and both groups received 1.5 g of TXA q12 h from postoperative Day 1 (POD1) to POD3. Haemoglobin (Hb), haematocrit (Hct), coagulation parameters, fibrinogen degradation product (FDP), and D-dimer (D-D) were recorded from PAD1 to POD3. HBL was calculated using the gross formula and recorded as the primary outcome. Result: In all-over analyses, the patients in Group B had lower perioperative HBL (on PAD3, POD1, and POD3), preoperative HBL (HBLpre), decline of haemoglobin (ΔHb-on PAD3), allogeneic blood transfusion (ABT) rate, FDP (on PAD3), and D-D (on PAD3) compared with Group A. No significant differences were exhibited in postoperative HBL (HBLpost) between the 2 groups. In subgroup analyses, for patients who received intervention within 24 h, the result is consistent with the whole. For patients who received intervention over 72 h of injury, there were no significant differences in perioperative HBL, ΔHb, ABT rate, FDP, and D-D between the 2 groups. There were no significant differences in APTT, PT, the rate of venous thromboembolism, wound complications, or 90-day mortality between the 2 groups. Conclusion: For elderly intertrochanteric fracture patients, early posttraumatic antifibrinolysis with multidose TXA is effective in reducing perioperative HBL, which mainly manifests as the reduction of preoperative HBL, especially for patients injured within 24 h. Application of TXA beyond 72 h of injury was ineffective.

## 1. Introduction

It is well known that elderly patients exhibit a high incidence of hip fracture owing to osteoporosis, and these fractures are increasing yearly. By 2050, there will be 6.3 million hip fractures worldwide annually [1]. Most intertrochanteric fractures need surgery for the purpose of avoiding immobility-related complications, walking ability, and diminished quality of life [2]. Despite great progress having been made in surgical techniques and perioperative management [3,4,5,6], perioperative mortality can still reach 0.8–2%, and 30-day and 1-year mortality can even reach 8% and 25%, respectively [7,8,9]. Research demonstrates that anaemia caused by hip fractures and subsequent surgery is an important risk factor for perioperative mortality, especially when Hb levels are below 80 g/L [10]. A study has shown that 44% of intertrochanteric fracture patients are diagnosed with anaemia on admission and 87% postoperatively [11]. As confirmed, perioperative anaemia is associated with obvious perioperative hidden blood loss (HBL) [12]. For intertrochanteric fracture patients, perioperative HBL includes posttraumatic HBL caused by the initial fracture and postoperative HBL resulting from surgery. Notably, according to research, posttraumatic HBL might account for more than 80% of perioperative total blood loss [13]. Since hyperfibrinolysis caused by initial trauma and surgery is an important risk factor for HBL [14], how to effectively implement antifibrinolytic therapy during the perioperative period, especially the period after trauma and before the operation, to reduce perioperative HBL in elderly intertrochanteric fracture patients, is worth further study.

As a representative antifibrinolytic agent, tranexamic acid (TXA) has been proven to be effective in reducing perioperative blood loss in patients with an intertrochanteric fracture without increasing the risk of venous thromboembolism (VTE) [14,15,16,17,18,19,20]. Mehmet Ekinci et al., identified that a single intravenous dose (15 mg/kg) of TXA intraoperatively could significantly reduce the HBL (582.3 ± 341.2 mL vs. 857.8 ± 493.1 mL) and ABT rate (8% vs. 23.5%) following PFN in elderly patients with intertrochanteric fracture, while it does not increase the risk of DVT or thromboembolic events [19]. Fenwick et al. had also demonstrated the efficacy and safety of a 1 g intravenous combined 1 g topical application of tranexamic acid for open reduction and nailing and a 1 g intravenous application for closed reduction with nailing in intertrochanteric fracture patients [20]. Yet, of note, the current widely used TXA regimens are mostly focused intraoperatively and postoperatively; in other words, this regimen is only effective for hyperfibrinolysis caused by surgery but provides a limited effect for hyperfibrinolysis caused by the initial trauma. Given the valid haemostatic effect of TXA, we speculate that TXA may have strong potential to reduce posttraumatic HBL caused by initial trauma. However, few studies have been conducted on the effectiveness of TXA in posttraumatic hyperfibrinolysis management and HBL reduction in patients with intertrochanteric fractures.

The main purpose of this study was to evaluate the effectiveness of posttraumatic antifibrinolytic therapy with multiple doses of IV-TXA in reducing posttraumatic HBL and perioperative HBL. We hypothesized that patients who received IV-TXA post-trauma injury but before surgery would have lower posttraumatic HBL and perioperative HBL than patients who received a placebo.

## 2. Materials and Methods

### 2.1. Study Design

This prospective, double-blinded, randomized controlled trial (RCT) strictly followed the Consolidated Standards of Reporting Trials (CONSORT) guidelines [21] and has been registered in the Chinese Clinical Trial Registry (ChiCTR2100048217). The study was performed according to the ethical standards of the Declaration of Helsinki of 1964, and approval was obtained from the Medical Ethics Committee of the First Affiliated Hospital of Chongqing Medical University (Chongqing, China) (Ethical NO. 2020-46-2). Written informed consent and research authorization were obtained from all participants.

The purpose of this trial is to confirm whether posttraumatic antifibrinolysis reduces posttraumatic HBL and perioperative HBL among elderly intertrochanteric fracture patients. All operations were performed by the same surgical team. All orthopaedists, nurses, enrolled patients, and data collectors were blinded until the end of the study.

### 2.2. Patients

From June 2021 to September 2022, patients aged over 65 years who suffered an intertrochanteric fracture were evaluated upon admission. The exclusion, inclusion, and termination criteria are tabulated in Table 1. After admission, all enrolled patients were randomly divided into Group A (control group) and Group B (posttraumatic TXA group) in a 1:1 ratio with the informed consent of the authorized clinician.

### 2.3. Randomization and Blinding

Enrolled patients were randomly divided into two groups in a 1:1 ratio. Group randomization was carried out during processing to control the potential heterogeneity among patients due to the changes in baseline characteristics such as injury time. SPSS version 24.0 (IBM Corporation, Armonk, NY, USA) was used for randomization, with block sizes of 2 and 6, and was stratified by injury time (<24 h, 24–72 h, and >72 h). Injury time was defined as the time between fracture onset and the first use of saline (NS) or TXA. The injury time was stratified based on the results of Tian et al. [13], which showed that there is an ongoing bleeding stage that may last for 72 h after fracture in elderly intertrochanteric fracture patients, and the lowest HB level is detected beyond 24 h after injury. The group data will be placed in an opaque sealed envelope. Only the nurses who did not participate in the trial were allowed to look up the enrolment and give the corresponding treatment, and the statisticians had the authority to check the registration and collect the population data. Until the final data analysis, the surgical team and the patient will be blinded to the interventions.

### 2.4. Study Interventions

Studies have verified that the administration of 15 mg/kg TXA twice daily intravenously is effective in reducing blood loss [22,23], and in consideration of few elderly people weighing more than 100 kg in China, we made the decision to take a dose of 1.5 g IV-TXA every 12 h.

Group A: From postadmission Day 1 (PAD1) to the day before surgery, 100 mL normal saline (NS) was used intravenously q12 h. From postoperative Day 1 (POD1) to POD3, 1.5 g of TXA + 100 mL of NS was injected q12 h.

Group B: From PAD1 to the day before surgery, 1.5 g of TXA + 100 mL of NS was injected q12 h. From POD1 to POD3, 1.5 g of TXA + 100 mL of NS was injected every 12 h. Both groups received 1.5 g of IV-TXA 30 min before surgery on the operation day and the same dose 12 h later.

### 2.5. Perioperative Management

Low molecular weight heparin was administered for VTE prophylaxis as long as there was no active haemorrhage (such as active ulcer) after admission until the day before surgery. The management of operation time in our centre conformed to the guidelines of the National Institute for Health and Care Excellence for hip fractures [24]. All operations were performed by experienced orthopaedic surgeons in the same hospital. All patients underwent surgery on an extension table with the same decubitus under general anaesthesia, and all of them were treated with a PFNA-II fixation device. There was no need for routine drainage tube placement. Low-molecular-weight heparin was administered 12 h after the operation and repeated at 24 h intervals for 35 days for perioperative VTE prophylaxis. Intravenous or oral NSAIDs in combination with patient-controlled intravenous analgesia (PCIA) were used to manage postoperative pain. Daily function training was performed under the assistance of a physiotherapist, including strength training, walking training, and active range of motion training. ABT is triggered by haemoglobin < 70 g/L or by symptomatic anaemia (such as paleness, palpitations, and dizziness) with 70 g/L < haemoglobin < 100 g/L.

### 2.6. Outcomes and Data Collection

Routine blood tests were performed on postadmission days (PADs) 1–3 and postoperative day (POD1) 1–3 to determine haematocrit (Hct) and haemoglobin (Hb) levels, and the coagulogram was used to monitor the fibrinolytic and coagulation indicators.

The primary outcome was the HBL, which was calculated by the daily monitored haematocrit (HCT) levels according to two formulas that had been reported previously [25,26].

1. Patient blood volume (PBV) = k1 × height^3^ (m) + k2 × weight (kg) + k3. (Female: k1 = 0.356, k2 = 0.03308, k3 = 0.1833, male: k1 = 0.3669, k2 = 0.03219, k3 = 0.6041.)
2. Total blood loss (TBL) = PBV × (Hct_0_ − Hct_n_)/Hct_0_, with HCT_0_ being the baseline level of HCT tested on admission and HCT_n_ being the HCT level tested on the nth day postadmission or postoperatively.
3. Dominant blood loss (DBL) = (the weight of the bloody gauze − the dry weight of the gauze) + (the volume collected by the suction bottle − the volume used to wash the surgical area).
4. HBL = TBL − DBL + ABT.

Preoperative HBL (HBL_pre_), which was caused by fracture, referred to the HBL on PAD3. Postoperative HBL (HBL_post_) caused by the operation referred to the HBL from PAD3 to POD3 and was calculated using the following formula: HBL_post_ = HBL − HBL_pre_.

The decrease in haemoglobin (ΔHb) was calculated according to the daily recorded Hb levels. The formula is as follows: ΔHb = Hb_0_ − Hb_n_, where Hb_0_ refers to the Hb level that was tested on the admission day, and Hb_n_ refers to the Hb level that was tested on the nth day postadmission or postoperatively. ABT events were monitored daily perioperatively.

The secondary outcomes were fibrinolysis and coagulation parameters, including fibrinogen degradation product (FDP), D-dimer (D-D), prothrombin time (PT), and activated partial thromboplastin time (APTT). Intramuscular vein thrombosis (IVT) and deep vein thrombosis (DVT) in the enrolled patients were screened by colour Doppler ultrasonography on PAD1 and POD3. The main criterion for ultrasound diagnosis of IVT and DVT is the inability to collapse the vein under probe pressure [27]. Wound complications (such as bleeding, local haematoma, superficial and deep infection), VTE (including DVT, IVT, and PE) rate, and mortality were monitored until POD90.

### 2.7. Sample Size Calculation

PPAS 11 (NCSS, LLC, Kaysville, UT, USA) software was used to calculate the sample size through a 2-sided, 2-sample *t*-test. The sample size requirement was based on the primary outcome of HBL determined by previous studies. The HBL in the TXA group was 210 mL (standard deviation [SD] 202), and that in the control group was 359 mL (SD 290) [16]. Setting the sample allocation ratio, significance level (α), and power (1 − β) as 1.0, 0.05, and 0.90, respectively, indicated that 40 patients were needed for subgroup analysis. To compensate for the expected dropouts (10%, according to our previous experiment), 44 patients were required for each subgroup. This indicated that a minimum of 15 patients per group would be required to achieve 90% power to detect the difference between the two groups of different interventions (NS or TXA).

### 2.8. Statistical Analysis

SPSS statistical software version 21.0 software (SPSS Inc., Chicago, IL, USA) was used for analysis. The quantitative parameters were expressed as the mean and standard deviation (SD), and the qualitative parameters were expressed as percentages or frequencies. Independent *t*-tests were used to analyse continuous variables such as HBL. The chi-square test was used for qualitative variables such as ABT rate. All hypotheses were estimated by 2-sided tests with a statistical significance set at a *p* value < 0.05.

## 3. Result

### 3.1. Participant Flow and Baseline Characteristics

**Figure 1:**
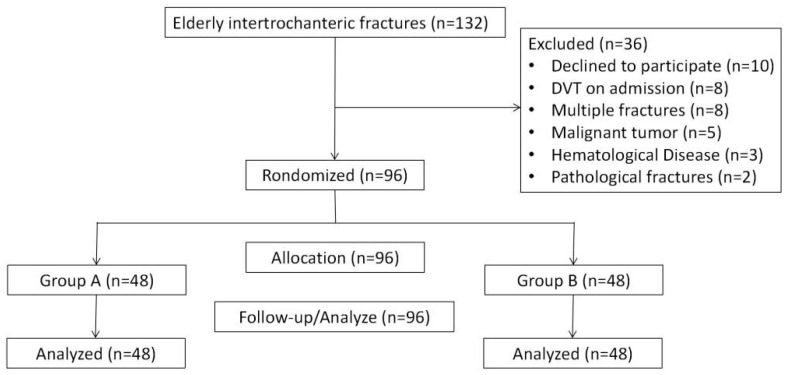
The flow chart of study enrolment. DVT, deep vein thrombosis.

Figure 1 demonstrates the enrolment and distribution of patients in this study. A total of 132 elderly patients with intertrochanteric fractures were treated in our centre between June 2021 and September 2022. Among these patients, 10 declined to participate, 8 were diagnosed with DVT on admission, 8 were complicated with multiple fractures, 5 were complicated with malignant tumours, 3 were combined with haematological disease, and 2 were pathological fractures. Finally, 96 patients were randomly divided into 2 different intervention groups at a ratio of 1:1, and the intervention groups were further divided into 3 subgroups in accordance with the injury time. The injury time ranged from 1 to 432 h in this trial. No significant difference was found between the two groups (Table 2). After meeting the minimum requirements of 15 patients in each subgroup, the trial was terminated. There was no loss of the 96 enrolled patients during the 90-day follow-up.

### 3.2. Primary Efficacy Outcomes

Details regarding the perioperative HBL, ΔHb, ABT rates and DBL are shown in Table 3. Compared with Group A, the patients in Group B had lower perioperative HBL on PAD3, POD1, and POD3 (*p* = 0.036, *p* = 0.026, and *p* = 0.040), lower ΔHb on PAD3 (*p* = 0.042), and lower ABT rates (*p* = 0.026). No significant differences were shown in DBL between the 2 groups. Group B had lower preoperative HBL (HBL_pre)_ than Group A (*p* = 0.036), but there was no significant difference in postoperative HBL (HBL_post_) between the 2 groups.

For patients who underwent intervention within 24 h after injury, the perioperative HBL on PAD2, PAD3, POD1, and POD3 was significantly lower in Group B than in Group A (*p* = 0.005, *p* = 0.016, *p* = 0.005, and *p* = 0.049). Consistent with the differences in perioperative HBL, ΔHb showed the same significant difference between the two groups on PAD3 (*p* = 0.016). The HBL_pre_ and ABT rates of Group B were also lower than those of Group A (*p* = 0.016, and *p* = 0.009). There were no significant differences in HBL_post_ and DBL between the 2 groups.

For patients who accepted intervention between 24 and 72 h after injury, compared with Group A, the patients in Group B showed lower perioperative HBL on POD1 and POD3 (*p* = 0.020, and *p* = 0.025), but for HBL on PAD3, there were no significant differences between the 2 groups. Similar to the differences in perioperative HBL, the ΔHb in Group B was lower than that in Group A on POD1, POD2, and POD3 (*p* = 0.007, *p* = 0.014 and *p* = 0.002), but no significant difference was exhibited on PAD3. There were no significant differences in HBL_post_, DBL, and ABT rates between the 2 groups.

For patients who accepted intervention over 72 h after injury, no significant differences were found in the perioperative HBL, DBL, ΔHb, and ABT rates of the 2 groups.

### 3.3. Secondary Efficacy Outcomes

**Figure 2:**
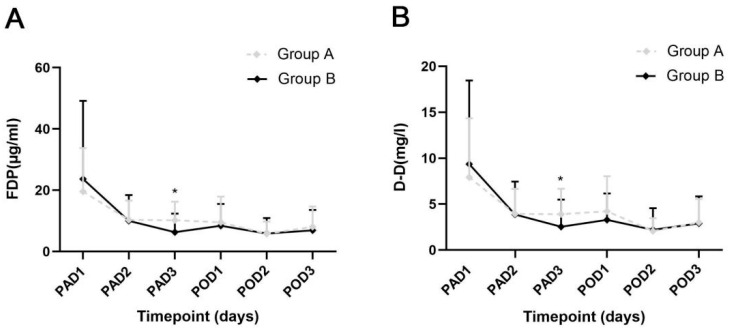
Dynamic changes of the 2 fibrinolysis parameters in integral analysis. (**A**) The levels of fibrinogen degradation product between the 2 groups. (**B**) The levels of D-Dimer between the 2 groups. PAD, postadmission day; POD, postoperative day; FDP, fibrinogen degradation product; D-D, D-dimer. Error bar, standard deviation; * *p* < 0.05.

**Figure 3:**
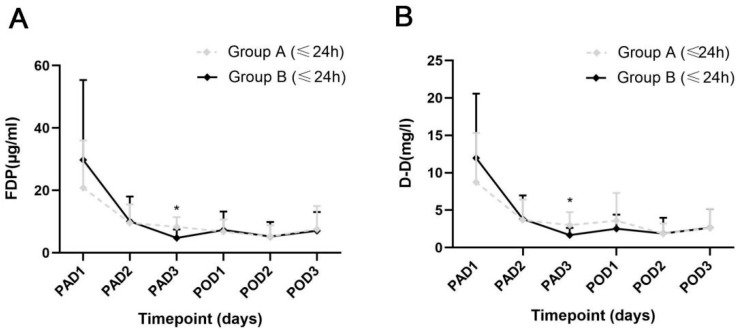
Dynamic changes of the 2 fibrinolysis parameters in the patients injured within 24 h. (**A**) The levels of fibrinogen degradation product between the 2 groups. (**B**) The levels of D-Dimer between the 2 groups. PAD, postadmission day; POD, postoperative day; FDP, fibrinogen degradation product; D-D, D-dimer. Error bar, standard deviation; * *p* < 0.05.

The specific values of D-D and FDP levels of the two groups are exhibited in Table 4, and the dynamic changes are shown in Figure 2. On PAD3, the patients in Group A exhibited lower levels of FDP and D-D than those in Group B (*p* = 0.008 and *p* = 0.049), which was consistent with the differences in perioperative HBL and ΔHb. On PAD1-2 and POD1-3, no significant differences were found in the FDP and D-D levels between the 2 groups. Similar results were exhibited in patients who accepted intervention within 24 h after injury (*p* = 0.004, and *p* = 0.019) (Figure 3).

**Figure 4:**
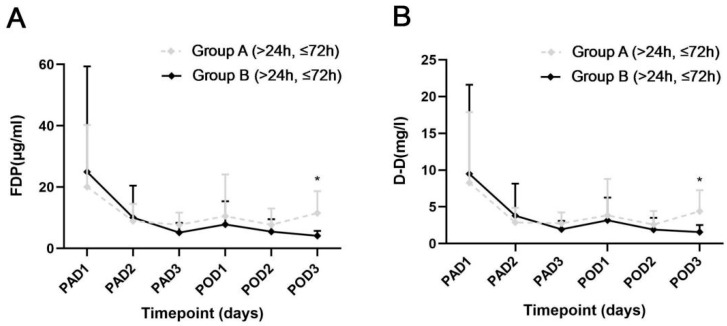
Dynamic changes of the 2 fibrinolysis parameters in the patients injured between 24 and 72 h. (**A**) The levels of fibrinogen degradation product between the 2 groups. (**B**) The levels of D-Dimer between the 2 groups. PAD, postadmission day; POD, postoperative day; FDP, fibrinogen degradation product; D-D, D-dimer. Error bar, standard deviation; * *p* < 0.05.

For patients who accepted intervention between 24 and 72 h after injury, compared with Group A, the patients in Group B had lower FDP and D-D levels on POD3 (*p* = 0.001, and *p* = 0.002), while on PAD1-3 and POD1-2, there were no significant differences (Figure 4).

**Figure 5:**
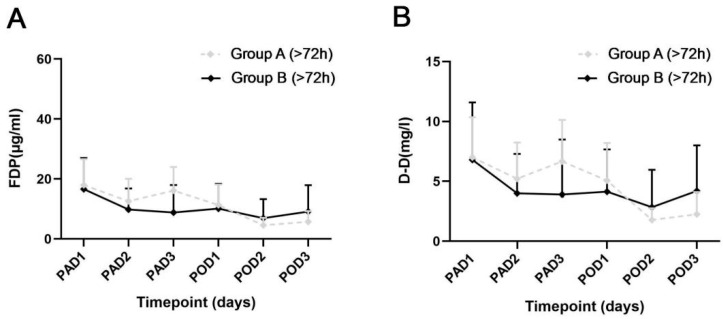
Dynamic changes of the 2 fibrinolysis parameters in the patients injured over 72 h. (**A**) The levels of fibrinogen degradation product between the 2 groups. (**B**) The levels of D-Dimer between the 2 groups. PAD, postadmission day; POD, postoperative day; FDP, fibrinogen degradation product; D-D, D-dimer. Error bar, standard deviation.

For patients who accepted intervention over 72 h after injury, no significant differences were found in the levels of FDP and D-D on PAD1-3 and POD1-3 (Figure 5).

The perioperative PT and APTT values were not significantly different between the 2 groups (Table 5), and the wound complications, length of stay, VTE rate, and 90-day mortality also did not show significant differences (Table 6).

## 4. Discussion

### 4.1. Main Findings

Our study found that (1) posttraumatic antifibrinolysis could further reduce perioperative HBL and ABT rates on the basis of the routine intraoperative and postoperative use of TXA in elderly intertrochanteric fracture patients. Furthermore, the decrease in perioperative HBL was mainly manifested as a decrease in preoperative HBL, while the postoperative HBL did not show significant changes; (2) for those patients who underwent intervention within 24 h, this antifibrinolytic therapy provided the best haemostatic effect, but for those who underwent intervention over 72 h, the haemostatic effect was unsatisfactory; and (3) no significant difference was found in the VTE rate in the present study.

### 4.2. Possible Mechanisms

Studies had identified that traumas including fractures would activate the fibrinolytic system of the body and lead to posttraumatic hyperfibrinolysis [14,28], which would aggravate the bleeding caused by trauma [29]. A previous study showed that preoperative HBL caused by initial fracture was up to 400 mL by Day 3 after fracture as a result of posttraumatic hyperfibrinolysis in intertrochanteric fracture patients [30]. As a haemostatic agent, TXA can effectively block fibrinolytic activity and prevent fibrin degradation by blocking the lysine-binding sites of plasminogen [31]. As a result, TXA effectively reduces posttraumatic HBL by inhibiting posttraumatic hyperfibrinolysis. In this study, the further reduction in perioperative HBL was mainly reflected in the reduction of preoperative HBL, which was precisely achieved by the posttraumatic antifibrinolysis effect of TXA, and this inhibition of posttraumatic hyperfibrinolysis could be demonstrated by the significantly lower D-D and FDP levels on PAD3 in the TXA intervention group. Similarly, in the subgroup analysis for patients injured within 24 h, the group that expressed significant fibrinolysis inhibition of PAD3 showed significant perioperative and preoperative HBL reduction. However, for patients injured over 72 h, this regimen could not provide effective posttraumatic inhibition of fibrinolysis; as a result, the TXA intervention group did not exhibit significant perioperative and preoperative HBL reduction.

Previous studies indicate that in intertrochanteric fracture patients, the posttraumatic hyperfibrinolysis will persist until 72 h after injury [13], and 72 h after injury may be the critical timepoint for effective early posttraumatic antifibrinolysis [32]. However, in the present study, we found an interesting phenomenon: in the subgroup of 24–72 h after injury, there was no significant decrease in preoperative HBL, FDP, and D-D levels of the TXA group on PAD3 compared with the control group, suggesting that TXA in this subgroup did not display a significant posttraumatic fibrinolytic inhibition effect. We speculate that there are two possible reasons: (1) The small sample size including 15 and 16 patients for each group may not be enough to reflect the significant differences in FDP and D-D levels between the two groups on PAD3. (2) This may be related to the fact that 72 h after injury is not the critical timepoint for effective early posttraumatic antifibrinolysis. Previous studies only confirmed that 72 h is the duration of HBL, but it cannot be equated with the effective time of antifibrinolytic therapy. Therefore, we speculate that there might be a time point between 24 and 72 h beyond which fibrinolysis inhibition is ineffective and the haemostatic effect of TXA is not ideal.

Due to its pharmacological mechanism, TXA is theoretically related to an increased risk of thrombosis. However, there is no direct evidence linking TXA to the increased risk of thrombosis thus far. In contrast, abundant clinical studies have confirmed the safety of the application of TXA, not only to patients undergoing elective TJA or TKA [33,34,35,36,37] but also to acute trauma patients [29], including hip fracture patients [14,15,16,17,18]. Similarly, the present study also did not find any increased risk of thrombosis in patients who received posttraumatic antifibrinolysis. On the one hand, the two major coagulation parameters (APTT and PT) were not significantly different from PAD1 to POD3 between the two groups; on the other hand, the VTE rate at the 90-day follow-up did not exhibit a significant difference between the two groups, and it had been confirmed that the 90-day follow-up was long enough to detect adverse events related to TXA because of the short half-life of this drug [38]. However, of note, due to the low incidence of VTE and the small sample size of this study, it might be too early to draw definitive conclusions. Therefore, a large-scale trial is required to complete the safety evaluation of this novel protocol.

### 4.3. Implications for Clinical Practice

A study confirmed that 44% of elderly intertrochanteric fracture patients had anaemia on admission, and after the operation, this rate increased to 87% [11], which is related to multiple adverse outcomes, including mortality [39]. Posttraumatic HBL induced by initial fracture is an important cause of perioperative anaemia. Smith demonstrated that the posttraumatic HBL was up to 400 mL by Day 3 after fracture [30]. Therefore, reducing posttraumatic HBL could be an important method to prevent perioperative anaemia. The present study showed that posttraumatic antifibrinolysis based on the routine use of TXA intraoperatively and postoperatively effectively reduced perioperative HBL and posttraumatic HBL. Hence, we recommend this novel TXA regimen to intertrochanteric fracture patients injured within 72 h to reduce posttraumatic HBL and perioperative HBL, especially the patients who are injured less than 24 h.

### 4.4. Call for Future Studies

Until now, a tremendous number of studies have inquired about the use of TXA intraoperatively and postoperatively in intertrochanteric fracture patients [14,15,16]. However, few studies have discussed the posttraumatic application of TXA, especially the administration time. The present study showed that administration within 24 h after injury provided the best haemostatic effect, but beyond 72 h after injury, it did not provide an encouraging haemostatic effect.

A previous study demonstrated that there is a potential time-related influence of preoperative HBL, and the ongoing bleeding stage is almost 72 h after intertrochanteric fracture [13]. This seems to indicate that TXA can provide a haemostatic effect as long as it is administered within 72 h after injury. However, of note, although the bleeding time for intertrochanteric fractures will last for 72 h after injury, it is unknown whether the best opportunity for effective anti-fibrinolytic therapy would be 72 h. In the present study, for patients who accepted intervention between 24 and 72 h after injury, the preoperative levels of FDP and D-D did not exhibit a significant reduction in the TXA intervention groups, which indicated that fibrinolysis inhibition did not work well at this timescale, and there should be another critical time point within 24–72 h, yet not at 72 h, beyond which antifibrinolysis therapy is ineffective and cannot reduce posttraumatic HBL. However, due to the limitation of sample size, we did not perform further stratification for patients injured between 24 and 72 h after injury, and the specific location of the critical time point is unknown.

In addition, few studies have included the onset treatment time of TXA in intertrochanteric fracture patients. The CRASH-2 trial recommended the administration of TXA within 3 h for bleeding trauma patients to reduce mortality [29]. Similarly, in patients with traumatic brain injury, administration of TXA within 3 h is recommended to exert its haemostatic effect [40]. The present study showed that TXA application within 24 h can provide the best haemostatic effect in intertrochanteric fracture patients. However, it is regrettable that we did not perform further stratification for this subgroup due to the limitation of the sample size; hence, it is not clear whether there is a better onset time of administration within 24 h after injury.

Therefore, there is a desperate need for further large-sample studies to confirm the optimal posttraumatic administration time of TXA in intertrochanteric fracture patients.

### 4.5. Strengths and Limitations

To our knowledge, this is the first study that applied posttraumatic antifibrinolysis based on the routine intraoperative and postoperative application of TXA in intertrochanteric fractures. The results confirmed that this novel dosage regimen is beneficial for reducing posttraumatic and perioperative HBL and should be recommended.

This study still had several limitations, although it had been carefully designed. First, the sample size was relatively small, and although the sample size calculations indicated that this was adequate, further research with a large sample size would be required to determine the best TXA regimen in patients with intertrochanteric fractures, especially the administration time. Second, because the calculation of the sample size was based on HBL, for other outcomes such as ΔHb, ABT rates, and VTE, it might be insufficient to draw specific conclusions. Third, the calculation of HBL was on the basis of HCT that was tested on the admission day rather than pre-trauma, and the HBL shown in the present study was ineluctably underrated because of the decrease in the HCT level. Finally, the postoperative dosage regimen of TXA was also novel, and the underlying differences in the outcomes might be covered up. Nevertheless, all patients in this study received the same postoperative dosage regimen, of which the haemostatic effect had been demonstrated by a previous study [22,23].

## 5. Conclusions

In summary, this small-sample RCT demonstrates that for elderly intertrochanteric fracture patients, posttraumatic antifibrinolytic therapy is an effective intervention to attain the further reduction of perioperative HBL, which mainly manifests as preoperative HBL reduction, especially among patients injured within 24 h. However, if the injury time is over 72 h, posttraumatic TXA administration cannot provide an effective haemostatic effect. Large-scale RCTs are required to determine the optimal time of this posttraumatic application of TXA and evaluate the safety of posttraumatic antifibrinolytic therapy in elderly intertrochanteric fracture patients.

## Figures and Tables

**Table 1:** Inclusion, Exclusion, and Termination Criteria.

Number	Criteria
	Inclusion
1	Aged over 65
2	Unilateral fresh intertrochanteric fracture
3	Receiving PFNA fixation
	Exclusion
1	Allergy to TXA
2	Combined with multiple fractures
3	Pathological fracture
4	Preoperative hepatic or renal dysfunction
5	Preoperative INR > 1.4, APTT > 1.4 * normal, platelets < 140,000/mm^3^
6	History of fibrinolytic disorder or Haematological Disease
7	History of DVT or PE
8	Cerebrovascular accident, myocardial infarction, New York Heart Association Class I or IV heart failure, atrial fibrillation
	Termination Criteria
1	Shock
2	Allergic symptoms
3	Reactive dermatitis, hypotension, dizziness, headache; vertigo, convulsions, blurred vision, etc.
4	Intracranial thrombosis and intracranial haemorrhage

PFNA, proximal femoral nail antirotation; TXA, tranexamic acid; INR, international normalized ratio; *, multiplied by; APTT, activated partial thromboplastin time; DVT, deep vein thrombosis; PE, pulmonary embolism.

**Table 2:** Comparison of Demographics by Study Group.

Demographics	Total			Injury Time ≤ 24 h			24 h < Injury Time ≤ 72 h			Injury Time > 72 h		
	Group A (*n* = 48)	Group B (*n* = 48)	*p* Value	Group A (*n* = 17)	Group B (*n* = 16)	*p* Value	Group A (*n* = 15)	Group B (*n* = 16)	*p* Value	Group A (*n* = 16)	Group B (*n* = 16)	*p* Value
Age, y (SD)	76.23 (7.96)	77.29 (8.23)	0.522 ^a^	77.59 (9.00)	77.31 (7.15)	0.923 ^a^	73.87 (7.20)	77.38 (8.33)	0.221 ^a^	77.00 (7.43)	77.19 (9.59)	0.951 ^a^
BMI, kg/m^2^ (SD)	22.43 (3.53)	21.60 (3.96)	0.284 ^a^	22.25 (2.89)	20.63 (2.86)	0.116 ^a^	23.40 (3.67)	21.46 (4.63)	0.209 ^a^	21.71 (4.00)	22.72 (4.14)	0.487 ^a^
Female gender, *n* (%)	29 (60.42%)	23 (47.92%)	0.219 ^b^	10 (58.83%)	9 (56.25%)	0.881 ^b^	8 (53.33%)	7 (43.75%)	0.594 ^b^	11 (68.75%)	7 (43.75%)	0.154 ^b^
Injury time, h (SD)	68.83 (88.61)	48.65 (51.65)	0.176 ^a^	10.35 (5.49)	8.50 (4.79)	0.311 ^a^	34.27 (9.56)	30.63 (4.81)	0.187 ^a^	163.38 (99.39)	106.81 (51.36)	0.052 ^a^
Operating time, min (SD)	85.17 (21.75)	91.63 (19.97)	0.133 ^a^	88.06 (24.54)	86.50 (18.85)	0.840 ^a^	83.13 (14.43)	93.56 (16.75)	0.074 ^a^	84.00 (25.12)	94.81 (23.89)	0.222 ^a^
LOS, d (SD)	10.65 (2.30)	10.08 (2.12)	0.214 ^a^	10.65 ()1.84	9.75 (1.61)	0.147 ^a^	11.53 (2.56)	10.63 (2.70)	0.346 ^a^	9.81 (2.32)	9.88 (1.86)	0.933 ^a^

SD, standard deviation; BMI, body mass index; LOS, length of stay. ^a^ Independent-samples *t*-tests. ^b^ Chi-squared test.

**Table 3:** Perioperative Bleeding Values and Allogeneic Blood Transfusion.

Outcome	Total			Injury Time ≤ 24 h			24 h < Injury Time ≤ 72 h			Injury Time > 72 h		
	Group A (*n* = 48)	Group B (*n* = 48)	*p* Value	Group A (*n* = 17)	Group B (*n* = 16)	*p* Value	Group A (*n* = 15)	Group B (*n* = 16)	*p* Value	Group A (*n* = 16)	Group B (*n* = 16)	*p* Value
HBL, mL (SD)												
PAD2	255.37 (440.51)	144.54 (312.10)	0.183 ^a^	440.47 (361.13)	103.28 (256.02)	0.005 ^a,c^	75.04 (573.83)	237.10 (406.27)	0.379 ^a^	194.39 (333.59)	81.41 (225.60)	0.322 ^a^
PAD3	363.72 (401.28)	187.18 (360.62)	0.036 ^a,c^	524.86 (388.12)	209.94 (312.80)	0.016 ^a,c^	352.89 (343.53)	230.54 (483.83)	0.494 ^a^	161.32 (390.78)	105.81 (227.08)	0.662 ^a^
POD1	652.27 (502.80)	434.65 (436.19)	0.026 ^a,c^	804.94 (466.19)	300.92 (499.75)	0.005 ^a,c^	760.71 (396.30)	432.18 (334.97)	0.020 ^a,c^	395.17 (547.20)	570.85 (441.96)	0.326 ^a^
POD2	758.97 (541.07)	568.39 (451.59)	0.088 ^a^	856.77 (564.12)	609.65 (411.80)	0.183 ^a^	812.70 (386.02)	480.13 (501.94)	0.082 ^a^	581.48 (623.52)	602.76 (467.69)	0.920 ^a^
POD3	646.50 (538.38)	437.76 (400.66)	0.040 ^a,c^	704.97 (517.05)	335.00 (502.11)	0.049 ^a,c^	759.25 (406.58)	428.70 (325.49)	0.025 ^a,c^	462.75 (657.42)	542.02 (348.59)	0.678 ^a^
POST-OP	214.71 (595.55)	174.60 (501.79)	0.745 ^a^	119.26 (714.87)	44.41 (555.72)	0.746 ^a^	432.87 (537.97)	91.04 (481.25)	0.116 ^a^	163.90 (410.17)	414.80 (396.57)	0.143 ^a^
ΔHB, g/L (SD)												
PAD2	8.64 (13.07)	5.00 (8.99)	0.145 ^a^	10.71 (11.21)	3.69 (8.36)	0.060 ^a^	7.89 (8.05)	7.93 (11.18)	0.993 ^a^	6.92 (17.62)	3.57 (6.93)	0.515 ^a^
PAD3	12.74 (14.58)	5.75 (9.81)	0.011 ^a,c^	17.75 (12.69)	7.53 (9.21)	0.016 ^a,c^	11.10 (10.21)	5.94 (12.65)	0.288 ^a^	7.85 (18.22)	3.46 (6.04)	0.418 ^a^
POD1	23.55 (19.80)	16.50 (12.86)	0.042 ^a,c^	27.06 (18.66)	14.69 (16.45)	0.053 ^a^	27.57 (12.98)	14.31 (11.77)	0.007 ^a,c^	16.31 (24.48)	20.50 (9.12)	0.526 ^a^
POD2	27.20 (19.74)	21.05 (13.60)	0.108 ^a^	27.53 (20.33)	23.79 (15.00)	0.572 ^a^	30.36 (9.72)	17.33 (13.08)	0.014 ^a,c^	24.08 (25.49)	21.50 (12.78)	0.740 ^a^
POD3	22.30 (19.33)	16.11 (14.78)	0.093 ^a^	17.75 (12.69)	7.53 (9.22)	0.305 ^a^	30.69 (13.36)	14.36 (11.65)	0.002 ^a,c^	14.21 (23.87)	18.00 (12.36)	0.583 ^a^
POST-OP	8.00 (17.10)	10.37 (18.09)	0.556 ^a^	4.63 (17.27)	8.00 (24.92)	0.650 ^a^	18.00 (16.48)	8.64 (16.09)	0.178 ^a^	3.82 (14.93)	14.77 (14.50)	0.083 ^a^
DBL, mL (SD)	197.81 (131.99)	195.62 (167.45)	0.943 ^a^	205.88 (115.76)	179.38 (186.71)	0.625 ^a^	169.67 (112.67)	179.38 (127.72)	0.824 ^a^	215.63 (165.04)	228.13 (187.06)	0.842 ^a^
ABT, *n* (%)	15 (32.61%)	6 (12.50%)	0.026 ^b,c^	8 (47.06%)	1 (6.25%)	0.009 ^b,c^	1 (6.67%)	3 (18.75%)	0.316 ^b^	6 (37.50%)	2 (12.50%)	0.102 ^b^

HBL, hidden blood loss; SD, standard deviation; PAD, postadmission day; POD, postoperative day; POST-OP, postoperative; ΔHB, decline in haemoglobin level; DBL, dominant blood loss; ABT, allogeneic blood transfusion. ^a^ Independent-samples *t*-tests. ^b^ Chi-squared test. ^c^ Statistically significant difference in mean values between groups.

**Table 4:** Perioperative Values of the 2 Main Fibrinolytic Parameters.

Outcome	Total			Injury Time ≤ 24 h			< 24 h Injury Time ≤ 72 h			Injury Time > 72 h		
	Group A (*n* = 48)	Group B (*n* = 48)	*p* Value	Group A (*n* = 17)	Group B (*n* = 16)	*p* Value	Group A (*n* = 15)	Group B (*n* = 16)	*p* Value	Group A (*n* = 16)	Group B (*n* = 16)	*p* Value
FDP, µg/mL (SD)												
PAD1	19.56 (14.24)	23.67 (25.50)	0.354 ^a^	20.81 (15.22)	29.77 (25.61)	0.254 ^a^	20.07 (20.19)	24.98 (34.38)	0.675 ^a^	17.96 (8.66)	16.64 (10.36)	0.698 ^a^
PAD2	10.33 (6.32)	10.01 (8.41)	0.861 ^a^	9.61 (5.90)	10.11 (7.90)	0.857 ^a^	8.90 (5.63)	10.10 (10.37)	0.765 ^a^	12.57 (7.49)	9.82 (7.00)	0.374 ^a^
PAD3	10.23 (6.05)	6.34 (6.04)	0.008 ^a,b^	8.34 (3.11)	4.80 (2.68)	0.004 ^a,b^	7.71 (3.96)	5.19 (3.15)	0.099 ^a^	16.15 (7.89)	8.85 (9.14)	0.070 ^a^
POD1	9.53 (8.39)	8.42 (7.09)	0.497 ^a^	6.78 (3.92)	7.30 (5.92)	0.753 ^a^	10.50 (13.63)	7.85 (7.53)	0.522 ^a^	11.31 (6.80)	10.12 (8.24)	0.664 ^a^
POD2	5.84 (3.95)	5.88 (5.05)	0.967 ^a^	5.28 (3.52)	5.22 (4.67)	0.972 ^a^	7.74 (5.28)	5.49 (4.01)	0.228 ^a^	4.57 (2.46)	6.93 (6.36)	0.222 ^a^
POD3	8.06 (6.62)	6.94 (6.64)	0.439 ^a^	7.55 (7.44)	7.08 (6.00)	0.852 ^a^	11.51 (7.19)	4.11 (1.62)	0.001 ^a,b^	5.71 (3.54)	9.13 (8.79)	0.186 ^a^
D-D, mg/L (SD)												
PAD1	7.93 (6.41)	9.36 (9.09)	0.393 ^a^	8.74 (6.58)	11.96 (8.62)	0.260 ^a^	8.28 (9.61)	9.49 (12.12)	0.785 ^a^	7.00 (3.36)	6.80 (4.80)	0.893 ^a^
PAD2	3.94 (2.72)	3.88 (3.58)	0.936 ^a^	3.69 (2.72)	3.83 (3.13)	0.904 ^a^	2.88 (1.99)	3.80 (4.35)	0.580 ^a^	5.22 (3.02)	4.01 (3.28)	0.377 ^a^
PAD3	3.91 (2.76)	2.54 (2.95)	0.049 ^a,b^	3.01 (1.72)	1.68 (0.95)	0.019 ^a,b^	2.76 (1.47)	1.94 (1.16)	0.152 ^a^	6.67 (3.46)	3.91 (4.58)	0.151 ^a^
POD1	4.22 (3.82)	3.29 (2.86)	0.186 ^a^	3.59 (3.71)	2.54 (1.87)	0.324 ^a^	3.84 (4.96)	3.18 (3.07)	0.673 ^a^	5.07 (3.14)	4.14 (3.53)	0.435 ^a^
POD2	2.07 (1.41)	2.21 (2.34)	0.741 ^a^	1.86 (1.36)	1.89 (2.09)	0.959 ^a^	2.60 (1.83)	1.91 (1.60)	0.312 ^a^	1.78 (0.97)	2.84 (3.13)	0.254 ^a^
POD3	3.01 (2.54)	2.91 (2.93)	0.873 ^a^	2.64 (2.54)	2.65 (2.50)	0.997 ^a^	4.41 (2.84)	1.58 (0.95)	0.002 ^a,b^	2.25 (1.86)	4.21 (3.79)	0.090 ^a^

FDP, fibrinogen degradation product; SD, standard deviation; PAD, postadmission day; POD, postoperative day; D-D, D-dimer. ^a^ Independent-samples *t*-tests. ^b^ Statistically significant difference in mean values between groups.

**Table 5:** Perioperative Values of Prothrombin Time and Activated Partial Thromboplastin Time.

Outcome	Total			Injury Time ≤ 24 h			< 24 h Injury Time ≤ 72 h			Injury Time > 72 h		
	Group A (*n* = 48)	Group B (*n* = 48)	*p* Value	Group A (*n* = 17)	Group B (*n* = 16)	*p* Value	Group A (*n* = 15)	Group B (*n* = 16)	*p* Value	Group A (*n* = 16)	Group B (*n* = 16)	*p* Value
PT, s (SD)												
PAD1	13.60 (0.93)	13.84 (1.00)	0.218 ^a^	13.67 (1.11)	13.85 (1.03)	0.636 ^a^	13.41 (0.89)	13.86 (1.05)	0.204 ^a^	13.70 (0.80)	13.81 (0.98)	0.725 ^a^
PAD2	13.67 (0.74)	13.65 (0.83)	0.890 ^a^	13.68 (0.56)	13.79 (0.81)	0.705 ^a^	13.56 (0.83)	13.71 (0.75)	0.632 ^a^	13.72 (0.86)	13.44 (0.96)	0.415 ^a^
PAD3	13.44 (1.09)	13.48 (0.93)	0.846 ^a^	13.43 (1.41)	13.49 (1.03)	0.881 ^a^	13.15 (0.56)	13.46 (0.74)	0.284 ^a^	13.67 (1.00)	13.49 (1.02)	0.636 ^a^
POD1	13.87 (0.92)	13.89 (0.97)	0.914 ^a^	13.78 (1.10)	13.93 (0.95)	0.670 ^a^	13.70 (0.65)	13.83 (1.19)	0.729 ^a^	14.11 (0.92)	13.91 (0.77)	0.528 ^a^
POD2	13.87 (0.88)	13.84 (1.04)	0.919 ^a^	13.75 (0.76)	13.78 (0.72)	0.924 ^a^	13.63 (0.65)	14.03 (1.37)	0.361 ^a^	14.22 (1.14)	13.71 (0.97)	0.222 ^a^
POD3	13.59 (1.14)	13.67 (1.47)	0.791 ^a^	13.72 (1.37)	13.48 (0.57)	0.549 ^a^	13.27 (0.67)	13.53 (1.42)	0.544 ^a^	13.75 (1.21)	13.92 (1.97)	0.783 ^a^
APTT, s (SD)												
PAD1	35.34 (4.06)	35.04 (3.98)	0.709 ^a^	34.91 (5.27)	34.65 (3.58)	0.870 ^a^	34.93 (3.52)	35.18 (5.38)	0.884 ^a^	36.16 (3.18)	35.28 (2.76)	0.409 ^a^
PAD2	37.27 (3.82)	35.60 (3.73)	0.054 ^a^	38.41 (4.48)	35.69 (3.78)	0.100 ^a^	36.93 (2.85)	36.31 (3.35)	0.644 ^a^	36.45 (3.76)	34.75 (4.13)	0.268 ^a^
PAD3	37.27 (4.08)	35.77 (4.00)	0.096 ^a^	37.81 (5.15)	36.52 (4.34)	0.454 ^a^	37.71 (3.99)	34.96 (4.05)	0.113 ^a^	36.31 (2.64)	35.74 (3.67)	0.647 ^a^
POD1	36.60 (3.80)	35.15 (3.16)	0.052 ^a^	36.44 (3.92)	35.58 (4.05)	0.548 ^a^	37.31 (4.65)	34.76 (2.95)	0.079 ^a^	36.03 (2.64)	35.11 (2.43)	0.320 ^a^
POD2	38.88 (4.56)	37.01 (4.52)	0.065 ^a^	39.01 (5.00)	37.95 (5.30)	0.571 ^a^	39.35 (5.56)	36.69 (4.64)	0.205 ^a^	38.30 (3.10)	36.40 (3.65)	0.159 ^a^
POD3	38.55 (5.51)	37.41 (4.55)	0.794 ^a^	38.26 (6.40)	36.34 (3.86)	0.347 ^a^	38.76 (5.07)	38.23 (4.84)	0.785 ^a^	38.67 (5.15)	37.68 (4.95)	0.596 ^a^

PT, prothrombin time; SD, standard deviation; PAD, postadmission day; POD, postoperative day; APTT, activated partial thromboplastin time. ^a^ Independent-samples *t*-tests.

**Table 6:** Comparison of Complications by Study Group.

Demographics	Total			Injury Time ≤ 24 h			<24 h Injury Time ≤ 72 h			Injury Time > 72 h		
	Group A (*n* = 48)	Group B (*n* = 48)	*p* Value	Group A (*n* = 17)	Group B (*n* = 16)	*p* Value	Group A (*n* = 15)	Group B (*n* = 16)	*p* Value	Group A (*n* = 16)	Group B (*n* = 16)	*p* Value
IVT on POD3, *n* (%)	6 (12.50%)	7 (14.58%)	0.765 ^a^	3 (17.65%)	2 (12.50%)	0.680 ^a^	0 (0.00%)	3 (18.75%)	0.078 ^a^	3 (18.75%)	2 (12.50%)	−9.626 ^a^
DVT on POD3, *n* (%)	4 (8.33%)	2 (4.16%)	0.399 ^a^	1 (5.88%)	0 (0.00%)	0.325 ^a^	2 (13.33%)	2 (12.50%)	0.945 ^a^	1 (6.25%)	0 (0.00%)	−9.310 ^a^
PE, *n* (%)	0 (0.00%)	0 (0.00%)	N/A	0 (0.00%)	0 (0.00%)	N/A	0 (0.00%)	0 (0.00%)	N/A	0 (0.00%)	0 (0.00%)	N/A
Would complications, *n* (%)	0 (0.00%)	0 (0.00%)	N/A	0 (0.00%)	0 (0.00%)	N/A	0 (0.00%)	0 (0.00%)	N/A	0 (0.00%)	0 (0.00%)	N/A
Death within POD90, *n* (%)	0 (0.00%)	0 (0.00%)	N/A	0 (0.00%)	0 (0.00%)	N/A	0 (0.00%)	0 (0.00%)	N/A	0 (0.00%)	0 (0.00%)	N/A

IVT, intramuscular venous thrombosis; POD, postoperative day; DVT, deep vein thrombosis; PE, pulmonary embolism; N/A, not applicable; ^a^ Chi-squared test.

## Data Availability

The data presented in this study are available in this article.
